# Determining the Protective Role of Grip Strength in Cardiovascular and Cerebrovascular Disease Risk Among Middle-Aged and Older Women in South Korea

**DOI:** 10.1007/s44197-026-00536-9

**Published:** 2026-03-20

**Authors:** En-Joo Jung, Mee-Ri Lee

**Affiliations:** 1 Department of Public Healthcare Center, Seoul National University Hospital, Seoul, Republic of Korea; 2 Department of Public Health and Medical Services, Seoul National University Hospital, Seoul, Republic of Korea; 3 Department of Preventive Medicine, Seoul National University College of Medicine, Seoul, Republic of Korea; 4 Department of Preventive Medicine, Soonchunhyang University College of Medicine, Cheonan, Republic of Korea

**Keywords:** Handgrip strength, Cardiovascular disease, Cerebrovascular disease, Older women, Longitudinal study, Aging

## Abstract

**Background:**

Handgrip strength is a simple measure of muscle function and health outcomes in older adults. Here, we investigated whether higher handgrip strength is associated with a lower risk of cardiovascular (CVD) and cerebrovascular (CeVD) diseases in middle-aged and older adults by focusing on its potential protective role in women.

**Methods:**

We analyzed data for approximately 9,500 participants of the Korean Longitudinal Study of Aging (aged ≥ 45 years, no CVD/CeVD at baseline). Handgrip strength was measured in multiple waves and treated as time-varying exposure. Cross-sectional logistic regression was used to evaluate baseline associations, while Cox regression models with time-varying handgrip strength were used to assess the 16-year incidences of CVD and CeVD after adjusting for major confounders. The analyses were stratified according to sex.

**Results:**

A higher handgrip strength was inversely associated with baseline CVD and CeVD prevalence (odds ratio, ~ 0.94–0.96 per 1-kg increase; *p* < 0.001). Over a 16-year follow-up, each 1-kg increase in handgrip strength predicted a lower hazard of CeVD (hazard ratio [HR], ~ 0.98) that was driven by a significant protective effect in women (HR, ~ 0.95) versus no association in men. Similarly, a higher handgrip strength predicted a lower CVD risk in women (HR, ~ 0.97) but not men.

**Conclusion:**

Greater handgrip strength was associated with lower risks of CVD and CeVD later in life. Although the associations appeared stronger in women, these differences should be interpreted cautiously given potential scaling effects. Handgrip strength may be a simple and clinically practical functional marker for vascular risk stratification in middle-aged and older adults.

**Supplementary Information:**

The online version contains supplementary material available at 10.1007/s44197-026-00536-9.

## Introduction

Handgrip strength has emerged as a significant health status assessment marker, particularly among older adults. Measuring handgrip strength is both simple and noninvasive, making it an attractive routine screening candidate in clinical and community settings. Its ease of use enables its widespread application without the need for sophisticated equipment or extensive training, which is crucial for large-scale public health initiatives targeting middle-aged and older populations [1, 2].

The prevalence of cardiovascular disease (CVD) and cerebrovascular disease (CeVD) in older populations is a significant public health concern, particularly as global demographics shift toward an aging society. Epidemiological data consistently demonstrate that disease incidence and burden increase with advancing age, with women representing a substantial proportion of affected individuals owing to their longer life expectancy than men [3].

Higher handgrip strength is associated with a lower risk of CVD in older women [2]. Recent research increasingly focused on its potential utility for predicting the risk of CVD and CeVD, especially in women who are at elevated risk due to age-related physiological changes [2]. Celis-Morales et al. [4] reported a 19% increase in cardiovascular mortality per 5-kg reduction in handgrip strength. This finding aligns with meta-analytic data indicating that each 1-kg increase in handgrip strength corresponds to a 29% reduction in cardiovascular mortality among older women [5]. Studies quantified a 15–20% risk increase per standard deviation (SD) decrease in handgrip strength [6] and demonstrated that a low handgrip strength is associated with a higher odd of dyslipidemia [7] and an elevated risk of arterial stiffness among women [8]. Thus, a lower handgrip strength is associated with increased mortality from both cardiovascular and non-cardiovascular causes [9]. Moreover, the stratification of participants according to handgrip strength revealed that women in the high stable handgrip strength group (30.3–32.4 kg) had more favorable baseline health characteristics and a lower prevalence of adverse cardiovascular outcomes than those in the low stable handgrip strength group (15.8–19.0 kg) [10].

In terms of cerebrovascular outcomes, studies reported a 54% higher risk of incident events (hazard ratio [HR] 1.54; 95% confidence interval [CI], 1.19–2.01) and a 1.6-fold higher stroke risk in women with lower handgrip strength [11, 12]. Another study revealed dose-response and threshold effects, with the risk increasing below specific relative handgrip strength cutoff points [13]. A study drawn from diverse cohorts across various age groups consistently indicated that a higher handgrip strength is associated with lower cardiovascular and cerebrovascular risks in older women [2].

While previous studies have confirmed associations between handgrip strength and cardiovascular/cerebrovascular outcomes, important gaps remain. First, handgrip strength represents a simple, accessible, and functional measure that could be implemented in community-based screening programs, yet its utility as a practical risk stratification tool for CVD and CeVD prevention has not been adequately established in Asian populations with long-term follow-up. Second, emerging evidence suggests sex-specific patterns in these associations, with some studies reporting stronger protective effects in women, although the mechanisms underlying these differences remain unclear. Third, systematic reviews and meta-analyses predominantly synthesized findings from Western cohorts, with limited representation from Asian populations in which CVD patterns and body composition characteristics may differ. For example, the large 17-country Prospective Urban Rural Epidemiology (PURE) study reported that every 5-kg decrease in handgrip strength corresponded to a 9% higher stroke risk and a 7% higher myocardial infarction risk, yet Asian populations remained underrepresented [14].

Therefore, this study aimed to separately determine the association between handgrip strength and the risk of CVD and CeVD using the Korean Longitudinal Study of Aging (KLoSA), which offers several unique advantages: [1] it is a nationally representative longitudinal panel survey with over 16 years of follow-up (nine waves), providing sufficient statistical power and temporal depth to examine long-term vascular outcomes; [2] handgrip strength was repeatedly measured across multiple waves, allowing the analysis of within-person changes over time rather than relying solely on baseline assessments; [3] the dataset includes comprehensive demographic and health information enabling robust adjustment for confounders; and [4] the large sample size permits sex-stratified analyses to explore differential associations between women and men. While KLoSA lacks biospecimen data, the availability of repeated functional assessments that can be easily implemented in clinical and community settings—such as handgrip strength—makes it particularly well-suited for evaluating pragmatic screening tools with direct public health applications. Furthermore, by examining CVD and CeVD as distinct outcomes rather than composite endpoints, this study provides more precise risk estimates for each vascular condition, enabling targeted prevention strategies with specific policy implications.

## Materials and Methods

### Study Design and Data Source

This study was based on data from KLoSA, an ongoing nationwide population-based cohort designed to be representative of community-dwelling middle-aged and older adults in South Korea (excluding Jeju). The KLoSA cohort was established in 2006 through stratified multistage probability sampling of individuals aged ≥ 45 years, enrolling approximately 10,254 participants in the first wave. To maintain representativeness, a cohort refresh was conducted in 2014 (wave 5) that added approximately 920 new participants, primarily those born in 1962–1963. For this study, two baseline entry points were defined.

Participants were eligible if they had no history of CeVD or CVD at baseline and complete data were available for all required covariates (Supplementary Figure S1). A schematic time-window diagram illustrating the alignment of exposure measurement and risk intervals is provided as Supplementary Figure S2. The final CeVD cohort comprised 9,553 participants (4,233 men, 5,320 women), while the CVD cohort comprised 9,497 participants (4,221 men, 5,276 women).

The study was approved by the Institutional Review Board of Soonchunhyang University (IRB no. SCH IRB 2025-09-014).

### Handgrip Strength

At each survey wave, handgrip strength (in kilograms) was measured twice using a handheld dynamometer (model NO6103; Tanita, Tokyo, Japan). In accordance with the standardized survey protocol, the maximal value obtained from the two trials was recorded as the handgrip strength of the participant for that wave. No additional transformation or derivation of the raw measurements was performed. Because handgrip strength was repeatedly assessed across waves, the analysis incorporated time-varying measurements, thereby reducing the potential influence of regression-to-the-mean effects. For the survival models, handgrip strength was treated as a time-varying covariate aligned with the inter-wave risk intervals. When the handgrip strength measure of a participant was missing in a later wave but available previously, we applied the last observation carried forward (LOCF) strategy for that individual. The participants for whom all eight wave-specific handgrip values were missing were excluded a priori. Absolute handgrip strength values (kg) at baseline by sex are shown in Supplementary Table S1.

### CVD and CeVD

CVD and CeVD were defined in this study as self-reported physician diagnoses of heart attack, angina, myocardial infarction, congestive heart failure, or other heart diseases and stroke, cerebral hemorrhage, cerebral infarction, or other cerebrovascular conditions reported since the last survey. For the time-to-event analyses, the event time was set to the month corresponding to the first wave in which the diagnosis was newly reported; otherwise, the observations were censored in December 2022. As the exact dates of diagnosis were unavailable, the event time was defined as the midpoint between the last disease-free interview and the diagnosis.

### Covariates

All models were adjusted for a consistent set of potential confounders measured at baseline. These included age (in years), sex (male or female), education level, marital status, smoking status, alcohol use, and body mass index (BMI). Education was treated as a categorical variable (≤ 6, 7–9, 10–12, or ≥ 13 years of education). Marital status was classified as married versus not married (including never married, divorced, or widowed). Smoking status was categorized as never smoker, former smoker, or current smoker. Alcohol consumption was coded as a binary indicator of current drinking status (yes or no). BMI was calculated from self-reported height and weight and treated as a continuous variable (expressed as kg/m²).

### Statistical Analysis

The baseline characteristics of the participants are summarized by sex. Continuous variables are presented as mean ± SD and compared using two-sample t-tests and categorical variables as n (%) and compared using χ² tests. As a pre-test, we fitted multivariable logistic regression models wherein the dependent variable was whether a participant ever reported receiving the diagnosis during the study period (yes/no) and the exposure was the mean handgrip strength across all observed waves; the models were run in the total sample and stratified according to sex. However, these models did not consider event timing.

We structured the data using inter-wave interval splitting to allow handgrip updates across survey waves. Follow-up time was divided into 2-year intervals aligned with the biennial survey schedule, and Cox proportional hazards models with time-varying covariates were fitted. Handgrip strength was updated at the start of each interval using the LOCF strategy. The Breslow method was used for analyzing ties and clustered robust standard errors in the models at the participant level. We fit the models in the total sample (including sex) first, followed by the sex-stratified models. Results are reported as HR and 95% CI values per 1-kg increase in handgrip strength. We visualized Kaplan–Meier curves according to handgrip tertile and present model-based adjusted survival curves.

Statistical significance was set at two-tailed values of *p* < 0.05. All analyses were conducted using Stata version 19 (StataCorp LLC, College Station, TX, USA) and R version 4.5.1 (R Foundation for Statistical Computing, Vienna, Austria) for graphical visualization.

## Results

### Baseline Characteristics of Participants

The baseline characteristics of the participants, stratified according to sex, are summarized in Table 1 (CeVD cohort) and 2 (CVD cohort).

**Table 1:** Sociodemographic and clinical characteristics of participants by sex (CeVD cohort)

Variable	Total (*N* = 9,553)	Male (*n* = 4,233)	Female (*n* = 5,320)	*P* value
**Age**,** y**,** mean ± SD**	59.8 ± 10.5	59.6 ± 10.1	59.9 ± 10.8	0.177
**Education**,** n (%)**				< 0.001*
≤6	3,841 (40.2)	1,159 (27.4)	2,682 (50.4)	
>6 but ≤ 9	1,531 (16.0)	677 (16.0)	854 (16.0)	
>9 but ≤ 12	2,945 (30.8)	1,512 (35.7)	1,433 (26.9)	
≥13	1,236 (12.9)	885 (20.9)	351 (6.6)	
**Marital status**,** n (%)**				< 0.001*
Married	7,740 (81.0)	3,902 (92.2)	3,838 (72.1)	
Not married	1,813 (19.0)	331 (7.8)	1,482 (27.9)	
**Smoking status**,** n (%)**				< 0.001*
Never smoker	6,843 (71.6)	1,687 (39.9)	5,156 (96.9)	
Former smoker	1,866 (19.5)	1,724 (40.7)	142 (2.7)	
Current smoker	844 (8.8)	822 (19.4)	22 (0.4)	
**Alcohol consumption**,** n (%)**				< 0.001*
Yes	3,880 (40.6)	2,782 (65.7)	1,098 (20.6)	
No	5,673 (59.4)	1,451 (34.3)	4,222 (79.4)	
**BMI**,** kg/m²**,** mean ± SD**	23.2 ± 2.7	23.2 ± 2.5	23.2 ± 2.9	0.762
≥25, n (%)	2,104 (22.0)	887 (20.9)	1,217 (22.9)	0.024*
**Handgrip strength**,** mean ± SD**				< 0.001*
Wave 1	26.7 ± 8.8	33.9 ± 7.1	21.0 ± 5.1	
Wave 2	26.0 ± 8.2	32.5 ± 6.6	20.7 ± 5.0	
Wave 3	25.3 ± 8.3	31.6 ± 6.9	20.1 ± 5.1	
Wave 4	25.9 ± 9.4	32.5 ± 9.0	20.6 ± 5.7	
Wave 5	27.2 ± 8.9	33.7 ± 7.7	22.0 ± 5.7	
Wave 6	27.5 ± 9.1	34.2 ± 7.7	22.1 ± 6.0	
Wave 7	26.8 ± 8.7	33.3 ± 7.5	21.7 ± 5.7	
Wave 8	26.2 ± 8.5	32.3 ± 7.6	21.4 ± 5.7	
**Hypertension**,** n (%)**	2,224 (23.3)	887 (20.9)	1,337 (25.1)	< 0.001*
**Diabetes**,** n (%)**	992 (10.4)	465 (11.0)	527 (9.9)	0.086

*BMI* body mass index, *CeVD* cerebrovascular disease, *SD* standard deviation

*p < 0.05. Values were derived from chi-squared tests for categorical variables and independent t-tests for continuous variables

**Table 2:** Sociodemographic and clinical characteristics of participants by sex (CVD cohort)

Variable	Total (*N* = 9,497)	Male (*n* = 4,221)	Female (*n* = 5,276)	*P* value
**Age**,** y**,** mean ± SD**	59.8 ± 10.5	59.6 ± 10.1	59.9 ± 10.8	0.244
**Education**,** n (%)**				< 0.001*
≤ 6	3,818 (40.2)	1,158 (27.4)	2,660 (50.4)	
> 6 but ≤ 9	1,521 (16.0)	675 (16.0)	846 (16.0)	
> 9 but ≤ 12	2,927 (30.8)	1,506 (35.7)	1,421 (26.9)	
≥ 13	1,231 (12.9)	882 (20.9)	349 (6.6)	
**Marital status**,** n (%)**				< 0.001*
Married	7,698 (81.1)	3,892 (92.2)	3,806 (72.1)	
Not married	1,799 (18.9)	329 (7.8)	1,470 (27.9)	
**Smoking status**,** n (%)**				< 0.001*
Never smoker	6,800 (71.6)	1,687 (40.0)	5,113 (96.9)	
Former smoker	834 (8.8)	813 (19.3)	21 (0.4)	
Current smoker	1,863 (19.6)	1,721 (40.8)	142 (2.7)	
**Alcohol consumption**,** n (%)**				< 0.001*
Yes	3,869 (40.7)	2,776 (65.8)	1,093 (20.7)	
No	5,628 (59.3)	1,445 (34.2)	4,183 (79.3)	
**BMI**,** kg/m²**,** mean ± SD**	23.2 ± 2.7	23.2 ± 2.5	23.2 ± 2.9	0.633
≥ 25, n (%)	2,090 (22.0)	889 (21.1)	1,201 (22.8)	0.047*
**Handgrip strength**,** mean ± SD**				< 0.001*
Wave 1	26.7 ± 8.8	33.9 ± 7.1	21.0 ± 5.1	
Wave 2	26.0 ± 8.2	32.5 ± 6.7	20.7 ± 5.0	
Wave 3	25.3 ± 8.3	31.6 ± 6.9	20.1 ± 5.1	
Wave 4	25.9 ± 9.4	32.5 ± 9.0	20.6 ± 5.7	
Wave 5	27.3 ± 8.9	33.8 ± 7.7	22.0 ± 5.7	
Wave 6	27.5 ± 9.0	34.2 ± 7.7	22.2 ± 6.0	
Wave 7	26.8 ± 8.7	33.3 ± 7.5	21.7 ± 5.7	
Wave 8	26.2 ± 8.5	32.3 ± 7.6	21.4 ± 5.7	
**Hypertension**,** n (%)**	2,201 (23.2)	883 (20.9)	1,318 (25.0)	< 0.001*
**Diabetes**,** n (%)**	975 (10.3)	458 (10.9)	517 (9.8)	0.093

*BMI* body mass index, *CeVD* cerebrovascular disease, *SD* standard deviation

*p < 0.05, †p < 0.10. Values were derived from chi-squared tests for categorical variables and independent t-tests for continuous variables

Among the 9,553 participants in the CeVD cohort, men had a significantly higher mean handgrip strength (all *p* < 0.001) and higher BMI (*p* < 0.05), whereas women were older on average (*p* < 0.05). Educational attainment, marital status, smoking status, alcohol consumption, overweight prevalence, hypertension prevalence, and exercise participation also differed significantly between the sexes (all p-values from the chi-square tests are shown in Tables 1 and 2).

### Cross-Sectional Analyses (Preliminary Logistic Regression)

In the CeVD cohort (Supplementary Table S1), higher handgrip strength was inversely associated with CeVD in the overall sample (odds ratio [OR], 0.94; 95% CI, 0.92–0.96) as well as in both men (OR, 0.94; 95% CI, 0.92–0.97) and women (OR, 0.92; 95% CI, 0.88–0.96). Similarly, in the CVD cohort (Supplementary Table S2), higher handgrip strength was associated with lower odds of CVD in the overall sample (OR, 0.96; 95% CI, 0.94–0.98), with consistent associations seen in men (OR, 0.96; 95% CI, 0.94–0.98) and women (OR, 0.94; 95% CI, 0.91–0.98).

### Time-to-Event Analyses

During a median follow-up of 16.3 years, 486 participants developed CVD and 411 developed CeVD.

In the CeVD cohort (Table 3), each 1-kg increase in handgrip strength was associated with a significantly lower hazard of CeVD in the overall sample (HR, 0.98; 95% CI, 0.96–0.99) and among women (HR, 0.95; 95% CI, 0.92–0.99) but not among men (HR, 0.99; 95% CI, 0.97–1.01).

In the CVD cohort (Table 4), a higher handgrip strength was protective in women (HR, 0.97; 95% CI, 0.95–0.999; *p* = 0.046), whereas the association was not statistically significant in men (HR, 0.99; 95% CI, 0.97–1.01) or the overall sample (HR, 0.99; 95% CI, 0.97–1.002).

**Table 3:** Handgrip strength and risk of incident cerebrovascular disease (Cox proportional hazards models by sex)

Variable	Total (*n* = 9,553) HR (95% CI)	Men (*n* = 4,233) HR (95% CI)	Women (*n* = 5,320) HR (95% CI)
**Handgrip strength (per 1-kg ↑)**	0.98 (0.96–0.99)*	0.99 (0.97–1.01)	0.95 (0.92–0.99)*
**Age**,** y**	1.03 (1.02–1.04)*	1.03 (1.02–1.05)*	1.02 (1.00–1.04)
**Female sex**	0.49 (0.36–0.68)*	—	—
**Education (ref: ≤6)**			
6–9	0.99 (0.73–1.34)	1.10 (0.73–1.65)	0.87 (0.55–1.38)
9–12	0.91 (0.68–1.21)	1.05 (0.72–1.54)	0.68 (0.42–1.09)
≥12	0.54 (0.35–0.84)*	0.59 (0.35–0.98)*	0.47 (0.18–1.22)
**Marital status (ref: Married)**	1.22 (0.91–1.65)	1.38 (0.75–2.54)	1.19 (0.82–1.73)
**Smoking status (ref: Never)**			
Former smoker	0.83 (0.63–1.11)	0.75 (0.54–1.04)†	1.85 (0.57–5.97)
Current smoker	0.91 (0.64–1.29)	0.88 (0.62–1.25)	—
**Alcohol consumption (ref: No)**	1.13 (0.89–1.43)	1.17 (0.87–1.56)	1.09 (0.72–1.63)
**BMI (kg/m²)**	1.07 (1.04–1.11)*	1.10 (1.05–1.15)*	1.04 (0.99–1.10)†

*BMI* body mass index, *CI* confidence interval, *HR* hazard ratio

*p < 0.05; †p < 0.10. — indicates not applicable or not estimable

BMI, body mass index; CI, confidence interval; HR, hazard ratio.

*p < 0.05; †*p* < 0.10. — indicates not applicable or not estimable.

**Table 4:** Handgrip strength and risk of incident heart disease (Cox proportional hazards models by sex)

Variable	Total (*N* = 9,497) HR (95% CI)	Male (*n* = 4,221) HR (95% CI)	Female (*n* = 5,276) HR (95% CI)
**Handgrip strength (per 1-kg ↑)**	0.99 (0.97–1.00)	0.99 (0.97–1.01)	0.97 (0.95–0.999)*
**Age**,** y**	1.02 (1.01–1.03)*	1.02 (1.01–1.04)*	1.02 (1.01–1.04)*
**Female**	0.90 (0.64–1.25)	—	—
**Education (ref: ≤6)**			
6–9	1.01 (0.77–1.33)	1.15 (0.76–1.76)	0.97 (0.67–1.41)
9–12	0.90 (0.70–1.16)	1.21 (0.83–1.76)	0.64 (0.42–0.97)*
≥12	0.88 (0.62–1.24)	1.03 (0.66–1.62)	0.72 (0.37–1.39)
**Marital status (ref: Married)**	1.69 (1.28–2.23)*	1.64 (0.91–2.96)†	1.91 (1.35–2.70)*
**Smoking status (ref: Never)**			
Former smoker	0.85 (0.64–1.13)	0.91 (0.67–1.23)	0.61 (0.33–1.15)
Current smoker	1.07 (0.77–1.50)	1.10 (0.78–1.56)	1.09 (0.25–4.70)
**Alcohol consumption (ref: No)**	1.01 (0.81–1.25)	1.11 (0.84–1.47)	0.89 (0.65–1.22)
**BMI (kg/m²)**	1.09 (1.06–1.12)*	1.10 (1.05–1.16)*	1.07 (1.03–1.11)*

*CI* confidence interval, *BMI* body mass index, *HR* hazard ratio

*p < 0.05; †p < 0.10. — indicates not applicable or not estimable

The proportional hazard assumption was evaluated using Schoenfeld residuals and showed no significant violations for any model.

### Survival Curves

The Kaplan–Meier survival curves for CeVD and CVD incidence stratified according to sex and handgrip strength tertiles are presented in Supplementary Figure S3.

## Discussion

In this nationally representative cohort of Korean adults aged ≥ 45 years with > 16 years of follow-up, a lower handgrip strength was independently associated with an elevated risk of both cardiovascular and cerebrovascular events after the adjustment for major confounders. Notably, stronger effect estimates per 1-kg increase were observed in women versus men. However, men had substantially higher absolute handgrip strength (mean, ~ 36 kg vs. ~22 kg in women), meaning a 1-kg increase might be suggestive of a smaller relative change in men (~ 2.8%) than women (~ 4.5%). Therefore, the larger HR in women may partly reflect measurement scale artifacts rather than true biological sex differences. Future studies should examine relative or standardized handgrip strength measures (e.g., normalized to body weight or z-scores) or formally test sex interactions.

These findings are consistent with those of previous studies demonstrating an inverse relationship between handgrip strength and CVD. The meta-analysis study by Wu et al. [15] showed that handgrip strength was inversely related to all-cause mortality and CVD incidence in community-dwelling populations; these associations remained significant after the adjustment for BMI, physical activity, serum cholesterol, smoking status, alcohol consumption, and socioeconomic status. Zhong et al. [11] observed higher risk profiles in groups with lower handgrip strength, aligning with the findings of Strand et al. [6] demonstrating that a substantial proportion of deaths were attributable to CVDs such as stroke and ischemic heart disease. Kumagai [16] demonstrated a clear linear dose-response relationship for both total and cardiovascular mortality. Sex-specific analyses revealed several important patterns, although these must be interpreted with the consideration of measurement scaling. Park and Park^1^ demonstrated that women in higher handgrip strength quartiles exhibited reduced ORs for atherosclerotic CVD, consistent with the findings of Song et al., [3] while Peralta et al. [2] identified critical sex-specific thresholds. The predictive capacity of weight-adjusted handgrip strength is particularly noteworthy, with Gu et al. [17] demonstrating a lower hypertension risk and Kim et al. [18] finding that obese women with a higher handgrip strength had lower all-cause mortality than non-obese women with a lower handgrip strength. Population-based studies consistently show higher mean age-adjusted handgrip strength values in men versus women [19], with mean values approximating 29.0 ± 6.3 kg in older women versus 45.4 ± 9.7 kg in men [20] and minimum recommended thresholds > 18.21 kg for older women [5].

Our study provides a unique perspective by separately examining handgrip strength in relation to CVD and CeVD outcomes in an Asian panel study with a particular focus on sex-stratified analyses. While CVD and CeVD share a vascular pathophysiology, we analyzed them as distinct outcomes because they represent different clinical entities with separate public health implications and preventive strategies. This approach allows for more precise risk stratification and targeted interventions specific to cardiac versus cerebrovascular health.

Previous research in predominantly Western populations consistently demonstrated associations between handgrip strength and cardiovascular events. The large 17-country PURE study reported that every 5-kg decrease in handgrip strength corresponded to a 9% higher stroke risk and 7% higher myocardial infarction risk over approximately 4 years [15]. A European cohort indicated that women in the highest handgrip strength quartile have an approximately 46% lower CVD incidence than those in the lowest quartile [2]. However, evidence from Asian cohorts is limited [21]. Our study helps bridge this gap by analyzing an aging East Asian population with a 16-year follow-up.

Our findings are largely in line with the emerging literature on handgrip strength and vascular health in Asian populations while contributing to the discussion on sex-specific patterns. Consistent with previous studies, we observed a protective association between handgrip strength and stroke risk, with stronger effect estimates in women. However, as noted above, this pattern should be interpreted cautiously considering the scaling differences between sexes. A large Chinese study found that a weak handgrip strength was associated with an 89% higher stroke risk over 4 years [21], while a South Korean analysis reported that a low relative handgrip strength was linked to an approximately threefold higher stroke odds in women [22]. The sex-specific patterns observed to date were not uniformly reported across all populations, and methodological factors such as absolute versus relative handgrip strength measures may have contributed to this heterogeneity. Women generally have a lower absolute muscle mass; therefore, a given decrease in absolute handgrip strength might signify a greater relative loss in physiological resilience among women versus men when expressed in absolute units. However, whether handgrip strength is truly more important for cardiovascular health in women or observed differences reflect scaling and body composition differences warrants further investigation using standardized measures. A statistically significant interaction between handgrip strength and sex was observed for CeVD, supporting stronger associations in women. In contrast, only a borderline interaction was observed for CVD, indicating that sex-specific differences may be more pronounced for cerebrovascular outcomes.

Asian studies indicated that low muscle strength can be at least as detrimental for cerebrovascular risk in women as in men [22]. Methodologically, rather than employing more complex full-likelihood interval-censored approaches [23], we conducted our primary analyses using extended Cox proportional hazards models that transparently and reproducibly incorporated handgrip strength as a time-varying exposure [24]. The association between handgrip strength and incident CVD was attenuated after the additional adjustment for cardiometabolic comorbidities (hypertension and diabetes), suggesting that part of the observed relationship may be explained by the underlying metabolic health of an individual. In contrast, the association with incident CeVD remained statistically significant despite full adjustment, indicating a more robust and potentially independent relationship (Supplementary Tables S4 and S5).

Handgrip strength serves as a multidimensional biomarker reflective of systemic physiological integrity. The association with cardiovascular outcomes is rooted in skeletal muscle functioning as a metabolic and endocrine organ [15], with age-related decline creating a cascade of physiological deteriorations that compromise cardiovascular health [25]. Importantly, because our models were adjusted for BMI, the observed associations reflect the effect of handgrip strength at constant BMI levels. Higher handgrip strength in this context likely indicates a more favorable body composition, i.e., greater muscle mass relative to fat mass, rather than merely reflecting the overall body size of an individual. Therefore, handgrip strength may function more as an integrated functional marker or risk stratification tool that captures multiple dimensions of physiological health than a single mechanistic exposure. This interpretation complements the mechanistic pathways discussed herein. The pathophysiology involves complex hormonal mechanisms, with age-related anabolic hormone decline occurring in both sexes, although women experience more abrupt reductions during menopause due to decreasing estrogen levels [11]. While the stronger effect estimates in women may partly reflect scaling artifacts, biological sex differences in hormonal regulation and body composition may also contribute.

Hao et al. [7] noted that genetic variations regulating muscle protein synthesis, mitochondrial function, and inflammatory responses may exert differential effects depending on hormonal milieu and sex. Lower handgrip strength correlates with higher hypertension and endothelial dysfunction rates [2], with sarcopenia (defined as a handgrip strength < 27 kg in men and < 16 kg in women) associated with adverse cerebrovascular outcomes [26]. Low-grade systemic inflammation is a critical mediator [27], with Laukkanen et al. [28] demonstrating that increased muscle strength facilitates more active lifestyles with lower chronic inflammation and reduced cardiovascular risk. Higher fitness correlates with reduced levels of the inflammatory markers implicated in cardiometabolic disorders [7]. Park et al. [29] suggested that resistance training may confer vascular benefits through enhanced endothelial responsiveness, increased shear stress, increased nitric oxide production, reduced proinflammatory cytokines, and improved insulin sensitivity, facilitating improved glucose metabolism and reduced insulin resistance.^1^ Socioeconomic factors further modulate these relationships, with lower income and education associated with reduced handgrip strength and poorer cardiovascular outcomes, particularly among women facing barriers such as limited healthcare access and elevated stress levels [1, 9]. Given that skeletal muscle is the largest organ system involved in glucose uptake, a diminished handgrip strength may reflect compromised glucose disposal capacity, potentially precipitating insulin resistance and diabetes [11], with reduced muscle mass correlated with a decreased basal metabolic rate and increased adiposity [13].

The implementation of handgrip strength as a screening tool is supported by its ease of measurement and strong prognostic value. Celis-Morales et al. [4] demonstrated that incorporating handgrip strength into office-based risk models significantly enhances the cardiovascular mortality prediction accuracy beyond traditional risk factors. Lin et al. [30] showed that handgrip strength serves as an indispensable marker for evaluating age-related declines in physical capacity. Our findings suggest that maintaining adequate handgrip strength is associated with lower cardiovascular and cerebrovascular risks in both women and men, although optimal absolute thresholds may differ between the sexes due to differences in muscle mass and body composition [9]. Intervention strategies targeting handgrip strength improvements operate through multiple pathways addressing underlying pathophysiological processes while modulating inflammation, metabolic dysfunction, and endothelial injury [19].

From a public health perspective, the handgrip strength assessment offers several advantages for population-level cardiovascular risk screening in that it: requires minimal equipment and training, can be rapidly administered in community settings, and provides objective functional assessment across diverse populations. Our finding that handgrip strength is independently associated with future CVD and CeVD events suggests its potential utility for targeting preventive interventions toward high-risk older adults before clinical disease onset. Specifically, routine handgrip strength screening in primary care or community health programs could identify individuals who would benefit from multicomponent interventions addressing sarcopenia, physical inactivity, and cardiovascular risk factors. Given the distinct policy implications of CVD versus CeVD prevention—including differential resource allocation for cardiac rehabilitation programs versus stroke prevention initiatives—separate risk assessments for these outcomes would enable more efficient public health resource deployment.

Interventional strategies targeting handgrip strength improvements operate through multiple pathways addressing underlying pathophysiological processes while modulating inflammation, metabolic dysfunction, and endothelial injury [19]. However, given our study design, these clinical implications should be considered preliminary, and they require validation through intervention studies. This study had several strengths, including featuring a large nationally representative cohort with a 16-year follow-up period, repeated handgrip strength measurements modeled as time-varying covariates, and the adjustment for multiple confounders. Nonetheless, this study had some limitations, including its observational design which precluded causal inferences and its use of self-reported physician diagnoses rather than medical record verification which may have introduced recall bias, misclassification, or underreporting. The lack of objective clinical validation (e.g., diagnostic codes, imaging results, or biomarkers) represents a significant limitation that could affect the precision of our effect estimates. Future studies should incorporate medical record linkage or objective diagnostic criteria to improve outcome ascertainment. Although we used the LOCF strategy to impute missing handgrip strength data, this method assumes that data are missing at random; in contrast, missingness related to health status could attenuate true associations.

An important methodological limitation is that we modeled handgrip strength in absolute terms rather than relative or standardized measures. This may have introduced scaling artifacts in the sex-stratified analyses, wherein 1-kg changes represent different relative magnitudes for women versus men. Future studies should normalize handgrip strength to body weight or height or use sex-specific z-scores. Additionally, while we adjusted for BMI to account for body size, handgrip strength at a constant BMI primarily reflects body composition (muscle-to-fat ratio) rather than a purely mechanistic exposure, which should be considered when interpreting the pathophysiological mechanisms. Furthermore, our models assumed a constant association between handgrip strength and cardiovascular/cerebrovascular risk across all ages despite the wide age range at baseline (45 + years). This assumption means that a single coefficient for handgrip strength is applied across all ages, but potential age-dependent heterogeneity in these associations remains unknown. Future studies should formally test for age interactions to determine whether the protective effects of handgrip strength vary across different age groups. Residual confounding due to unmeasured multimorbidity, medication use (including polypharmacy), orthopedic conditions, and cognitive impairment may have influenced the observed associations. Thus, future studies incorporating more detailed clinical and functional assessments are warranted. Finally, because our cohort consisted exclusively of older Korean adults, the generalizability of our findings to other populations requires replication.

In conclusion, maintaining adequate handgrip strength is strongly associated with the determinant of cardiovascular and cerebrovascular health in both women and men. Incorporating handgrip strength assessments into routine clinical evaluations is a practical way to enable early detection and personalized prevention. Future research should employ both absolute and relative handgrip strength measures, formally test for sex interactions, and develop sex-specific thresholds that account for differences in body composition. López-Bueno et al. [31] emphasized that sex-specific approaches may improve the identification of at-risk individuals, although this approach requires careful consideration of sex-specific thresholds, the use of standardized protocols, and the inclusion of potentially relative or normalized handgrip strength measures. Given these socioeconomic and geographic disparities, stratified longitudinal study designs are needed to develop tailored interventions that advance precise prevention and cardiovascular equity [6].

## Supplementary Information

Below is the link to the electronic supplementary material.

Supplementary Material 1

## Data Availability

The data supporting the findings of this study are publicly available from the Korean Longitudinal Study of Aging conducted by the Korea Employment Information Service (https://survey.keis.or.kr/klosa/klosadownload/List.jsp). The data are freely available without registration or application, and the authors did not have any special access privileges.

## Abbreviations

BMI: Body mass index
CeVD: Cerebrovascular disease
CI: Confidence interval
CVD: Cardiovascular disease
HR: Hazard ratio
KLoSA: Korean Longitudinal Study of Aging
LOCF: Last observation carried forward
OR: Odds ratio
SD: Standard deviation
