# Supplementary material for: Determining the Protective Role of Grip Strength in Cardiovascular and Cerebrovascular Disease Risk Among Middle-Aged and Older Women in South Korea
Source: J Epidemiol Glob Health. 2026 Mar 20;16(1):48. doi: 10.1007/s44197-026-00536-9 (PMC13057047; doi:10.1007/s44197-026-00536-9)
Supplement: Supplementary file 1 — Supplementary Material 1 [file 44197_2026_536_MOESM1_ESM.docx]

**
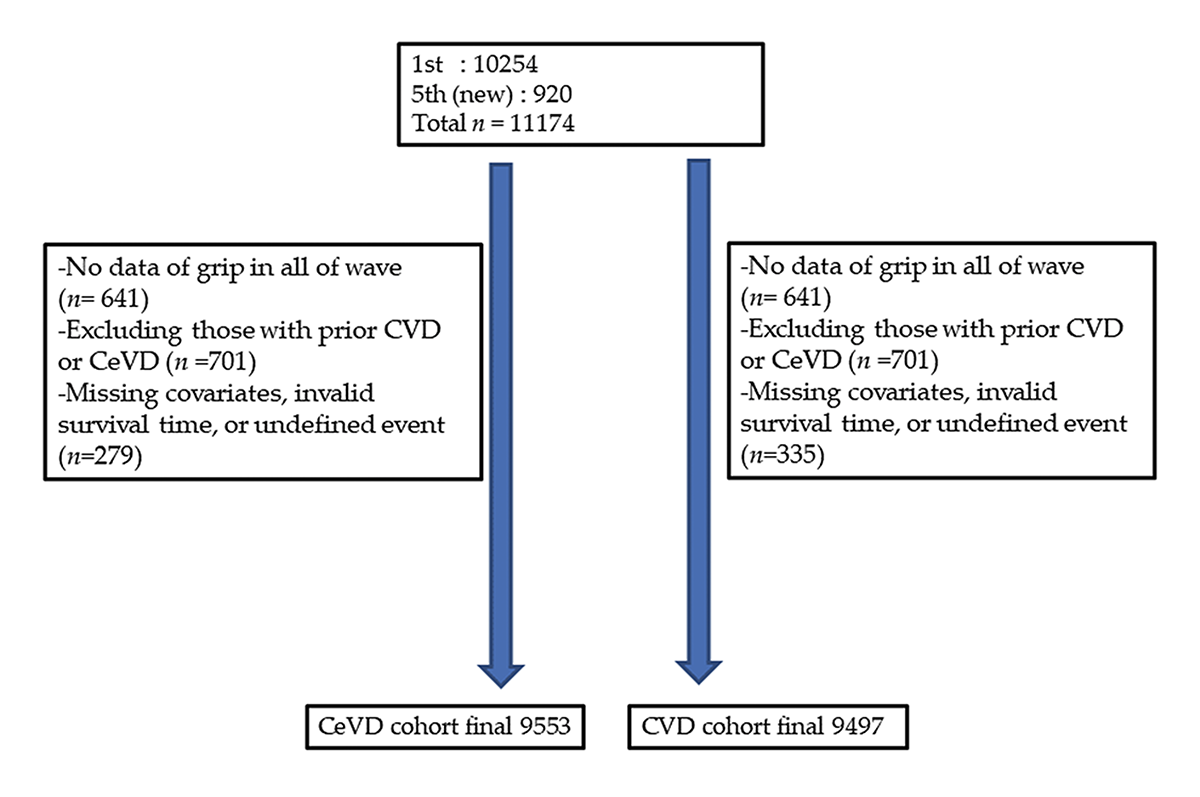
**

**Supplementary Figure S1.** Flowchart of participant selection for the CVD and CeVD cohorts.

CVD, cardiovascular disease; CeVD, cerebrovascular disease.


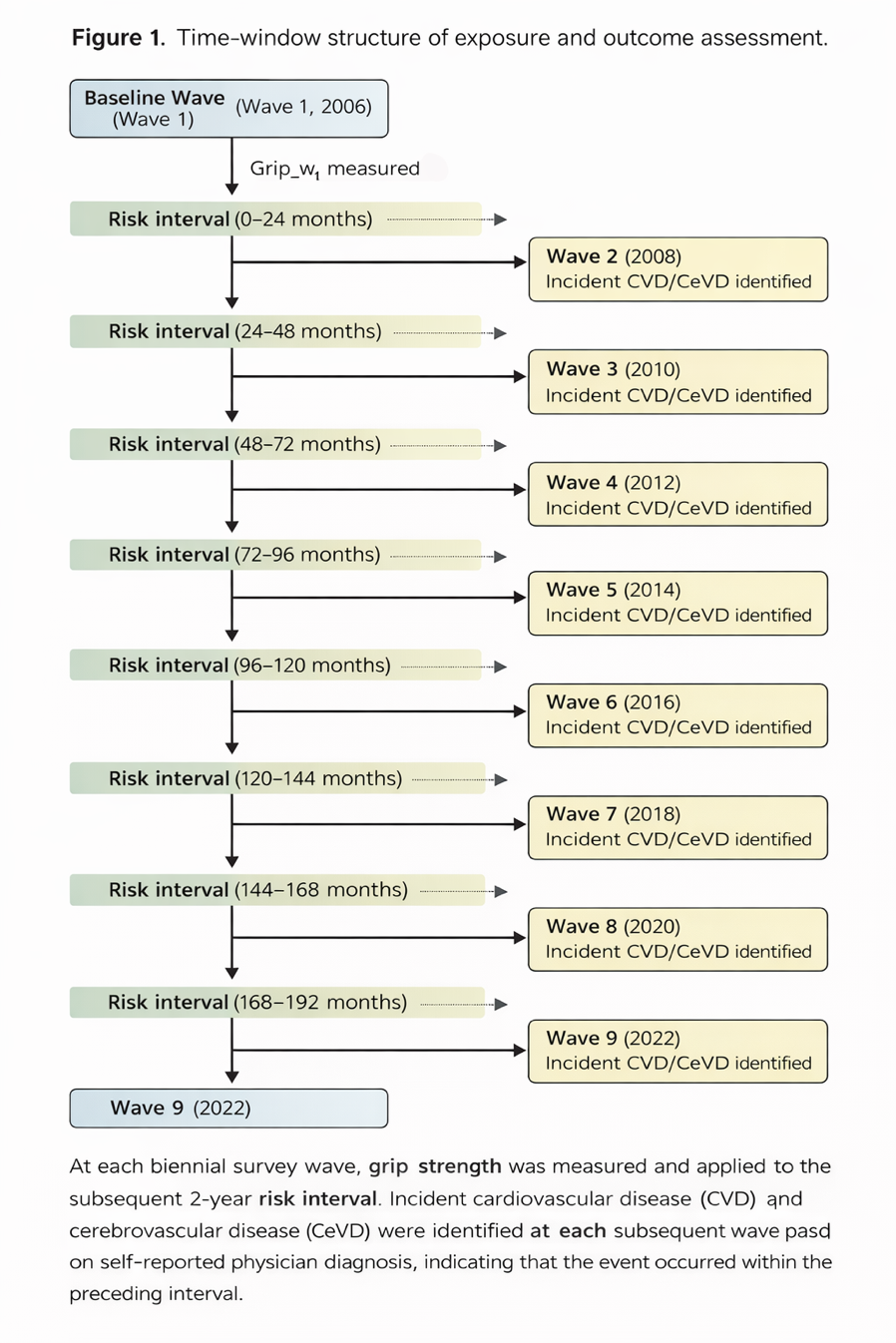


Supplementary Figure S2 Time-window structure of exposure and outcome assessment.


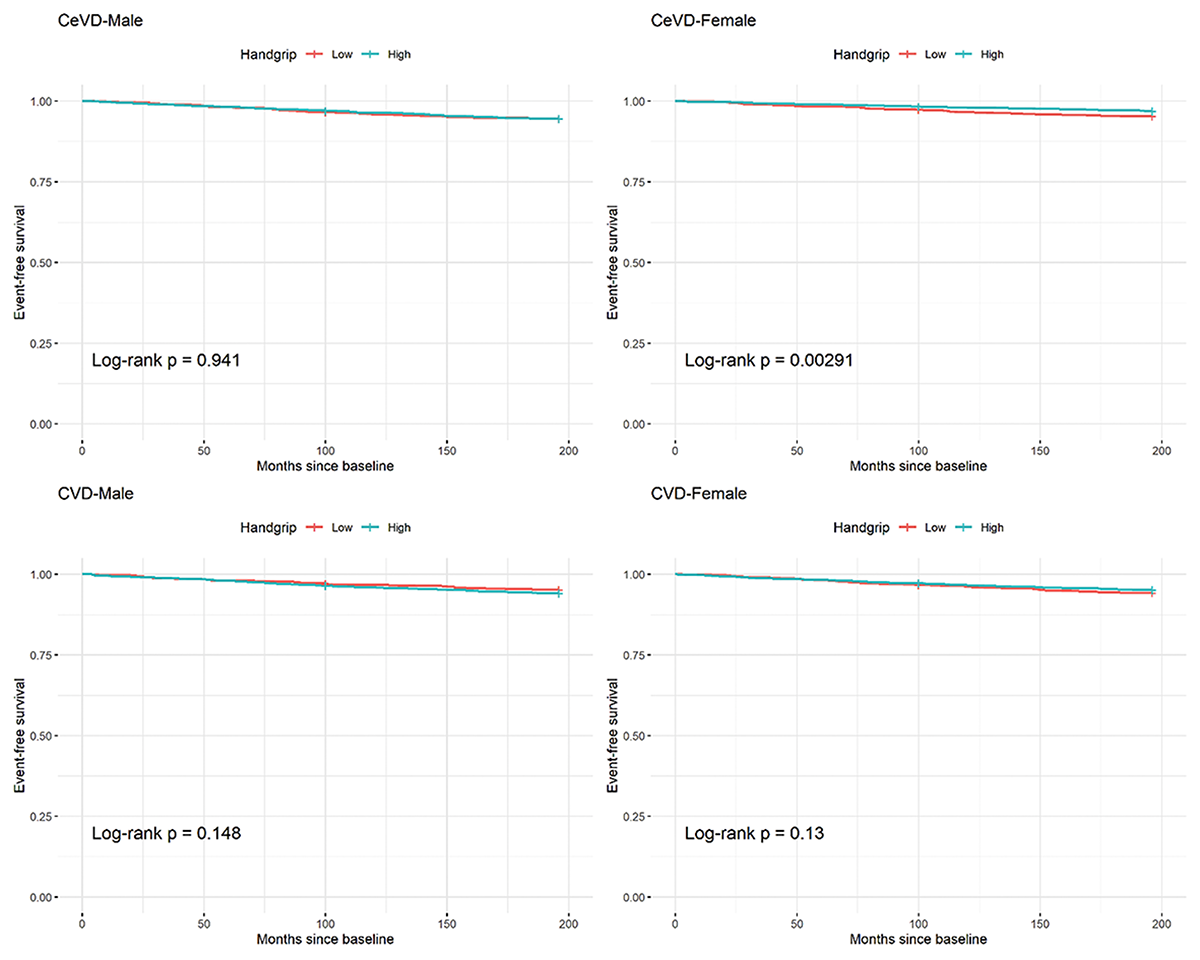


**Supplementary Figure S3.** Combined Kaplan–Meier survival curves for CVD and CeVD outcomes according to grip strength.

CVD, cardiovascular disease; CeVD, cerebrovascular disease.

Supplementary Table S1. Absolute Handgrip Strength (kg) at Baseline by Sex

| **Outcome dataset** | **Group** |  | **Mean ± SD (kg)** | **Median (IQR) (kg)** |
| --- | --- | --- | --- | --- |
| **CeVD cohort** | Men |  | 34.56 ± 7.17 | 35 (30–40) |
|  | Women |  | 21.69 ± 5.49 | 21 (19–25) |
|  | Total |  | 27.49 ± 8.98 | 26 (20–34.7) |
| **CVD cohort** | Men |  | 34.17 ± 7.18 | 35 (30–39) |
|  | Women |  | 21.24 ± 5.39 | 21 (18–25) |
|  | Total |  | 26.99 ± 8.96 | 25 (20–34) |

Abbreviations. IQR, interquartile range; SD, standard deviation.

**Supplementary Table S2.** Association between mean grip strength and heart disease events (logistic regression, CeVD cohort).

| **Variable** | **Total (n=9,553)**  **OR (95% CI)** | **Men (n=4,233)**  **OR (95% CI)** | **Women(n=5,298)**  **OR (95% CI)** |
| --- | --- | --- | --- |
| **Grip strength (per 1 kg ↑)** | 0.94 (0.92–0.96)* | 0.94 (0.92–0.97)* | 0.92 (0.88–0.96)* |
| **Age, y** | 1.02 (1.00–1.03) | 1.02 (1.00–1.04)† | 1.01 (0.99–1.03) |
| **Female** | 0.32 (0.22–0.47)* | – | – |
| **Education (ref: ≤6)** |  |  |  |
| 7–9 | 1.00 (0.74–1.35) | 1.13 (0.75–1.70) | 0.87 (0.55–1.38) |
| 10–12 | 0.94 (0.71–1.25) | 1.12 (0.78–1.61) | 0.67 (0.42–1.08)† |
| ≥13 | 0.55 (0.36–0.85)* | 0.60 (0.36–0.99)* | 0.51 (0.20–1.31) |
| **Married** | 1.29 (0.96–1.72) | 1.67 (0.93–2.98) | 1.20 (0.84–1.73) |
| **Smoking status (ref: Never)** |  |  |  |
| Former smoker | 0.83 (0.62–1.12) | 0.74 (0.54–1.03)† | 1.86 (0.58–5.95) |
| Current smoker | 0.92 (0.64–1.33) | 0.89 (0.62–1.29) | — |
| **Alcohol consumption (Yes)** | 1.11 (0.88–1.41) | 1.16 (0.86–1.55) | 1.06 (0.70–1.61) |
| **BMI (kg/m²)** | 1.08 (1.05–1.12)* | 1.12 (1.06–1.18)* | 1.05 (1.00–1.10) |

CeVD, cerebrovascular disease; OR, odds ratio; CI, confidence interval; BMI, body mass index.

* p < 0.05; † p < 0.10. — indicates not applicable or not estimable.

**Supplementary Table S3.** Association between mean grip strength and heart disease events (logistic regression, CVD cohort).

| **Variable** | **Total (n=9,497)**  **OR (95% CI)** | **Men (n=4,221)**  **OR (95% CI)** | **Women (n=5,276)**  **OR (95% CI)** |
| --- | --- | --- | --- |
| **Grip strength (per 1 kg ↑)** | 0.96 (0.94–0.98)* | 0.96 (0.93–0.99)* | 0.94 (0.91–0.98)* |
| **Age, y** | 1.02 (1.01–1.03)* | 1.01 (1.00–1.03) | 1.02 (1.00–1.03) |
| **Female** | 0.67 (0.47–0.96)* | – | – |
| **Education (ref: ≤6)** |  |  |  |
| 6–9 | 1.03 (0.78–1.35) | 1.20 (0.79–1.84) | 0.97 (0.67–1.42) |
| 9–12 | 0.88 (0.68–1.14) | 1.24 (0.86–1.79) | 0.60 (0.40–0.90)* |
| ≥13 | 0.86 (0.61–1.23) | 1.04 (0.67–1.62) | 0.69 (0.35–1.36) |
| **Married** | 1.72 (1.31–2.27)* | 1.68 (0.92–3.06)† | 1.99 (1.43–2.77)* |
| **Smoking status (ref: Never)** |  |  |  |
| Former smoker | 0.83 (0.62–1.11) | 0.89 (0.65–1.22) | 0.59 (0.31–1.13) |
| Current smoker | 1.09 (0.77–1.54) | 1.12 (0.78–1.61) | 1.18 (0.24–5.83) |
| **Alcohol consumption (Yes)** | 1.00 (0.80–1.24) | 1.09 (0.82–1.46) | 0.89 (0.64–1.24) |
| **BMI (kg/m²)** | 1.10 (1.07–1.14)* | 1.12 (1.06–1.18)* | 1.08 (1.04–1.13)* |

CVD, cardiovascular disease; OR, odds ratio; CI, confidence interval; BMI, body mass index.

* p < 0.05; † p < 0.10.

Supplementary Table S4. Grip strength and risk of incident CeVD (Cox proportional hazards models by sex).

| Variable | Total (n=9,553) | Men (n=4,233) | Women (n=5,320) |
| --- | --- | --- | --- |
| Grip strength (per 1 kg ↑) | 0.98 (0.96–0.99)* | 0.99 (0.97–1.01) | 0.95 (0.91–0.99)* |
| Age, y | 1.02 (1.01–1.03)* | 1.02 (1.00–1.04)* | 1.01 (0.99–1.03) |
| Female (vs male) | 0.44 (0.33–0.60)* | — | — |
| Education (ref: lowest) |  |  |  |
| edu_2 | 0.98 (0.72–1.33) | 1.06 (0.70–1.60) | 0.90 (0.56–1.43) |
| edu_3 | 0.95 (0.71–1.26) | 1.08 (0.74–1.57) | 0.69 (0.42–1.13) |
| edu_4 | 0.65 (0.40–1.04)† | 0.74 (0.43–1.28) | 0.43 (0.16–1.18) |
| Married | 1.20 (0.89–1.61) | 1.42 (0.77–2.59) | 1.22 (0.84–1.78) |
| Alcohol (yes) | 1.08 (0.86–1.36) | 1.10 (0.83–1.46) | 1.09 (0.72–1.64) |
| BMI (kg/m²) | 1.05 (1.02–1.09)* | 1.07 (1.02–1.12)* | 1.03 (0.98–1.08) |
| Occupation (ref: reference group) |  |  |  |
| Service/Sales | 2.05 (1.07–3.91)* | 2.60 (1.10–6.13)* | 0.85 (0.33–2.24) |
| Agriculture/Fishery | 2.37 (1.21–4.65)* | 3.50 (1.51–8.15)* | 0.88 (0.29–2.67) |
| Manual labor | 2.13 (1.18–3.84)* | 3.41 (1.66–7.01)* | 0.47 (0.17–1.31) |
| Unemployed | 2.20 (1.21–4.00)* | 3.43 (1.60–7.35)* | 0.76 (0.31–1.86) |
| Region (ref: metropolitan) |  |  |  |
| Mid-sized city | 1.48 (1.18–1.86)* | 1.45 (1.06–1.97)* | 1.52 (1.07–2.14)* |
| Rural | 1.16 (0.86–1.56) | 1.14 (0.75–1.74) | 1.18 (0.76–1.81) |
| Hypertension | 0.92 (0.87–0.98)* | 0.90 (0.83–0.97)* | 0.96 (0.88–1.04) |
| Diabetes | 0.95 (0.88–1.02) | 0.97 (0.88–1.07) | 0.93 (0.83–1.03) |

Abbreviations: HR, hazard ratio; CI, confidence interval; BMI, body mass index.

* p < 0.05; † p < 0.10.

Models were adjusted for age, education, marital status, alcohol consumption, BMI, occupation, region, hypertension and diabetes. Robust standard errors were clustered by participant ID (pid). Hazard ratios (HRs) with 95% confidence intervals (CIs) are presented.

## Supplementary Table S5. Grip strength and risk of incident CVD (Cox proportional hazards models by sex)

| Variable | Total (n=9,497) | Men (n=4,221) | Women (n=5,276) |
| --- | --- | --- | --- |
| Grip strength (per 1 kg ↑) | 0.99 (0.97–1.00) | 0.99 (0.97–1.01) | 0.97 (0.95–1.00)† |
| Age, y | 1.02 (1.01–1.03)* | 1.02 (1.00–1.03)† | 1.02 (1.00–1.04)* |
| Female (vs male) | 0.82 (0.61–1.09) | — | — |
| Education (ref: lowest) |  |  |  |
| edu_2 | 1.00 (0.76–1.32) | 1.12 (0.73–1.71) | 0.99 (0.68–1.43) |
| edu_3 | 0.94 (0.72–1.23) | 1.23 (0.84–1.79) | 0.68 (0.44–1.06) |
| edu_4 | 1.01 (0.71–1.43) | 1.19 (0.76–1.85) | 0.83 (0.43–1.60) |
| Married | 1.68 (1.27–2.22)* | 1.72 (0.95–3.12)† | 1.84 (1.30–2.59)* |
| Alcohol (yes) | 0.99 (0.79–1.22) | 1.09 (0.82–1.44) | 0.86 (0.62–1.19) |
| BMI (kg/m²) | 1.07 (1.04–1.10)* | 1.08 (1.03–1.14)* | 1.05 (1.01–1.09)* |
| Occupation (ref: white-collar) |  |  |  |
| Service/Sales | 1.57 (0.97–2.54)† | 1.65 (0.90–3.06) | 1.62 (0.63–4.15) |
| Agriculture/Fishery | 1.32 (0.77–2.27) | 0.88 (0.44–1.77) | 2.34 (0.84–6.51) |
| Manual labor | 1.67 (1.09–2.57)* | 1.80 (1.11–2.93)* | 1.43 (0.55–3.69) |
| Unemployed/Inactive | 1.40 (0.92–2.15) | 1.60 (0.94–2.73)† | 1.35 (0.56–3.29) |
| Region (ref: metropolitan) |  |  |  |
| Mid-sized city | 1.08 (0.88–1.33) | 1.16 (0.85–1.58) | 1.00 (0.75–1.34) |
| Rural | 1.17 (0.91–1.50) | 1.48 (1.04–2.12)* | 0.93 (0.65–1.32) |
| Hypertension | 0.90 (0.85–0.94)* | 0.71 (0.51–0.97)* | 0.60 (0.45–0.79)* |
| Diabetes | 0.91 (0.86–0.97)* | 0.68 (0.47–0.99)* | 0.76 (0.53–1.08) |

Abbreviations: HR, hazard ratio; CI, confidence interval; BMI, body mass index.

* p < 0.05; † p < 0.10.

Models were adjusted for age, sex (total model only), education, marital status, alcohol consumption, BMI, occupation, region, hypertension, and diabetes. Smoking was excluded in this sensitivity analysis.

Robust standard errors were clustered by participant ID (pid). Hazard ratios (HRs) with 95% confidence intervals (CIs) are presented.
